# Psychosocial risks at work: integrative review and conceptual perspectives

**DOI:** 10.47626/1679-4435-2025-1464

**Published:** 2025-07-13

**Authors:** Iara do Nascimento Teixeira, Isabel Soares Silva, Irene Maria Dias Cadime

**Affiliations:** 1 Escola de Psicologia, Universidade do Minho, Braga, Portugal; 2 Centro Interdisciplinar de Ciências Sociais (CICS.NOVA.UMinho); Escola de Psicologia, Universidade do Minho, Braga, Portugal; 3 Centro de Investigação em Estudos da Criança, Universidade do Minho, Braga, Portugal

**Keywords:** psychosocial risks, occupational health, occupational stress, integrative review, work psychology, riscos psicossociais, saúde ocupacional, estresse ocupacional, revisão integrativa, psicologia do trabalho

## Abstract

This study presents an integrative review of the scientific literature on psychosocial risks in the workplace. Its aim is to critically examine existing definitions, identify conceptual convergences and divergences, and propose a more comprehensive and operational understanding of the phenomenon. A total of 24 empirical articles published between 2017 and 2021 were analyzed from the Web of Science and Scopus databases, following the Preferred Reporting Items for Systematic Reviews and Meta-Analyses guidelines. The analysis revealed significant conceptual variation, reflecting diverse theoretical and contextual perspectives. A frequent confusion was observed between the terms “psychosocial risks” (referring to negative health outcomes) and “psychosocial risk factors” (the specific conditions that lead to those outcomes), which complicates practical implementation. Thus, an integrative definition was developed, emphasizing the complex interaction among structural, organizational, social, and individual conditions as well as subjective and contextual elements. The study also proposes a practical approach to operationalize this definition, integrating validated quantitative tools (e.g., Copenhagen Psychosocial Questionnaire, Job Content Questionnaire, iWorkHealth) with qualitative methods such as interviews and focus groups. The importance of clearly distinguishing between psychosocial risks, risk factors, and protective factors is underscored to support more effective organizational interventions. This review offers a significant contribution to the conceptual and operational clarity surrounding psychosocial risks, laying a solid foundation for practical application and fostering improvements in occupational health and workplace well-being.

## Abstract (pt)

Este estudo realizou uma revisão integrativa da literatura científica sobre riscos psicossociais no trabalho, visando analisar criticamente as definições existentes, identificar convergências e divergências conceituais e propor uma compreensão ampliada e operacional do fenômeno. Foram analisados 24 artigos empíricos publicados entre 2017 e 2021 nas bases Web of Science e Scopus, seguindo diretrizes Preferred Reporting Items for Systematic Reviews and Meta-Analyses. As análises revelaram uma significativa diversidade conceitual, refletindo diferentes abordagens teóricas e contextuais. Identificou-se também uma confusão frequente entre os termos “riscos psicossociais” (consequências negativas à saúde) e “fatores de risco psicossociais” (condições específicas que levam a essas consequências), dificultando a operacionalização prática. Como resultado, foi construída uma definição integradora, destacando a interação complexa entre condições estruturais, organizacionais, sociais e individuais, bem como fatores subjetivos e contextuais. Além disso, propõe-se uma operacionalização prática dessa definição, combinando métodos quantitativos validados (ex. Copenhagen Psychosocial Questionnaire, Job Content Questionnaire, iWorkHealth) com métodos qualitativos, como entrevistas e grupos focais. Ressalta-se a importância da diferenciação clara entre riscos psicossociais, fatores de risco e fatores protetores para facilitar intervenções eficazes nas organizações. Esta revisão contribui significativamente para a clareza conceitual e operacional dos riscos psicossociais, oferecendo bases sólidas para sua aplicação prática e melhorando potencialmente a saúde ocupacional e o bem-estar no ambiente laboral.

## INTRODUCTION

Work has long been a central organizing activity in human life, not only providing the means for subsistence but also offering a space for socialization, identity construction, and the development of interpersonal relationships.^1^,^2^ Recent changes in work dynamics have triggered significant shifts, intensifying both physical and psychological demands. These transformations have also undermined collective work practices, encouraged individualism, and contributed to burnout — all of which negatively impact health.^3^-^5^ At the heart of these changes lies the growing concern over psychosocial risks at work, which have become a priority area for researchers and policymakers due to their direct impact on occupational health and safety.

In Brazil, psychosocial risks are gaining increasing attention, particularly after Ordinance No. 1,419 from the Ministry of Labor and Employment included psychosocial risk factors as mandatory elements in the NR-1 Risk Management Program. This highlights the urgent need for clear conceptual definitions and practical strategies to ensure their effective management.^6^

Although psychosocial risks are widely recognized as critical challenges, defining and effectively managing them remains a complex task. Literature highlights difficulties inherent to their multifactorial nature since these risks involve psychological, social, and organizational factors, with implications that vary depending on the context and individual characteristics of workers.^7^ Additional obstacles in regulating and implementing effective policies — particularly at the European and international levels — further contribute to serious shortcomings in the prevention and mitigation of these risks.^8^,^9^

The precise definition of psychosocial risks in the workplace is a well-documented challenge in both the scientific literature and organizational practice. This difficulty stems from the complex and multifaceted nature of the concept, which includes psychological, social, organizational, and even cultural dimensions that vary according to context and individual worker experiences.^7^ The International Labour Organization (ILO) was one of the first institutions to acknowledge the urgent need to address psychosocial risks, yet it has faced significant challenges in defining the term since it was first formally recognized in 1984.^10^

A significant gap in conceptual clarity persists in the ongoing debates around psychosocial risks, making effective management challenging.^11^ These differences are often exacerbated by widespread confusion in the workplace between the terms psychosocial risks and psychosocial risk factors. Psychosocial risks refer to the likelihood that conditions in the work environment may lead to adverse effects on workers’ physical or mental health. In contrast, psychosocial risk factors are the specific job or organizational characteristics — such as excessive workloads, job insecurity, or interpersonal conflict — that contribute to these risks.^8^ This conceptual distinction is crucial for developing effective prevention and mitigation strategies. However, it is frequently overlooked in both research and practice. As a result, psychosocial risks are often narrowly interpreted as occupational stress or burnout, the latter being just one possible manifestation of these broader risks.^12^

Moreover, the assessment of psychosocial risks tends to be more subjective than that of other workplace hazards, which makes their management more complex. Workers may interpret and respond to the same risk factors in very different ways, depending on individual characteristics, coping abilities, and the level of organizational support they receive. Research shows that organizational support, trust, and work engagement play a key role in reducing psychosocial risks and fostering effective coping strategies.^13^ Additionally, interactions between individuals and their environments can lead to substantial variation in behavioral and psychological responses to workplace stressors, further reinforcing the need for integrated and personalized approaches.^14^ From this perspective, both working conditions and individual differences must be carefully considered in efforts to mitigate psychosocial risks.

Despite advances in the understanding of psychosocial hazards — and the elements associated with them — the ambiguity of the construct and ongoing terminological confusion continue to pose major challenges to the adoption of effective management practices in the workplace. The present study conducts an integrative literature review aimed at critically analyzing existing definitions of psychosocial risks at work, identifying points of convergence and divergence, and proposing a clear, operational conceptual framework.

## METHODS

An integrative literature review was conducted to examine how psychosocial risks in the workplace have been conceptualized. This type of review was selected because it allows for the synthesis of diverse methodological approaches — quantitative, qualitative, and conceptual — thereby supporting a critical and comprehensive analysis of the development of the concept. The review followed the methodological framework proposed by Whittemore & Knafl,^15^ which includes the following stages: formulation of the research question, development of inclusion and exclusion criteria, systematic database searches, quality assessment of selected studies, data extraction and categorization, and categorical synthesis leading to an integrative conceptual construction.

The search for research studies covered the period from 2017 to 2021, following the guidelines outlined in the Preferred Reporting Items for Systematic Reviews and Meta-Analyses (PRISMA) statement.^16^ PRISMA provides a framework to ensure transparency and methodological rigor in the conduct and reporting of systematic reviews — from formulating the research question to synthesizing results.

The protocol adopted for this review included the search strategy, inclusion and exclusion criteria, study selection process, and data analysis, as detailed in the following sections. This review aims to answer the review question: “What definitions of the concept of ‘psychosocial risks’ at work are found in recent literature?”

### ELIGIBILITY CRITERIA

To guide article selection, clear inclusion and exclusion criteria were established. Studies were included if they were peer-reviewed, written in Portuguese, English, or Spanish, and presented a definition of psychosocial risks at work. Excluded from the review were articles in other languages, studies without full-text access, non-peer-reviewed publications (e.g., dissertations, theses, and conference proceedings), and nonempirical works (e.g., essays).

### INFORMATION SOURCE, SEARCH STRATEGY, AND STUDY SELECTION

Searches were conducted in two major databases within the field of psychology: Web of Science and Scopus. The exact search string used for Web of Science was: psychosocial risks AND work (Title) OR psychosocial risks AND occupational (Title) AND 2017, 2018, 2019, 2020, or 2021 (Publication Years). For Scopus, the search string was: TITLE (psychosocial AND risks AND work) OR TITLE (psychosocial AND risks AND occupational) AND PUBYEAR > 2016 AND PUBYEAR < 2022.

The search results were managed using EndNote software and imported into the Covidence platform, where the stages of the integrative review were conducted. Initially, duplicate abstracts were removed. Two independent reviewers then evaluated the remaining abstracts, voting for inclusion or exclusion based on the established criteria. Any disagreements were resolved through discussion. This same procedure was applied to the full-text screening of articles selected during the abstract review phase.

After the final selection of articles, based on the eligibility criteria, the study proceeded to the final phase: data extraction and analysis.

### DATA EXTRACTION AND INFORMATION SYNTHESIS PROCESS

Data extraction was carried out independently by two reviewers using pre-defined criteria. The extracted information included general details (e.g., author, year, and title), study objectives, methodologies used, and results relevant to the topic. This information was then summarized to support further analysis focused on aspects such as general characteristics, purpose, methodology, and findings of each study. Definitions of psychosocial risks found in the selected articles were categorized into conceptual groups based on the theoretical authors cited. The categorization process was also conducted independently by two researchers. Any disagreements were resolved through discussion and consensus, enhancing the reliability of the classification.

Data analysis followed a categorical synthesis approach, as outlined by Torraco,^17^ grouping the identified definitions into conceptual categories based on the theoretical references cited in the studies. This method is well-suited for integrative reviews with a conceptual focus, as it enables the identification of convergences, divergences, and gaps in the current understanding of the analyzed concept.

### CONSTRUCTION OF THE INTEGRATING DEFINITION

The conceptual definition presented in this study was developed using the qualitative methodology of integrative conceptual synthesis.^15^,^17^ This process involved the following steps:

#### Identification of definitions

The definitions previously identified in the literature were examined and grouped according to conceptual themes and the theoretical orientations of the cited authors. The categorization process is detailed in the *Results* section of this study.

#### Critical comparative analysis

A comparative analysis was conducted across the identified categories, highlighting points of convergence and divergence between classical and contemporary authors.^18^-^21^ This step allowed the identification of conceptual strengths as well as limitations in the existing definitions.

#### Conceptual integration

A new integrative definition was then developed to address key shortcomings, particularly theoretical fragmentation and the lack of practical operationalization.

To ensure the robustness of this new definition, it incorporated recurring themes found across the reviewed literature, including multidimensionality (encompassing both objective and subjective components), the interactive nature of individual and contextual factors, and explicit recognition of both risks and protective factors.

The definition was further refined through collaborative discussion and consensus among the researchers involved in the analysis. Special consideration was given to the criteria of clarity, applicability, and alignment with contemporary international guidelines.

## RESULTS

### STUDY SELECTION

**Figure 1:**
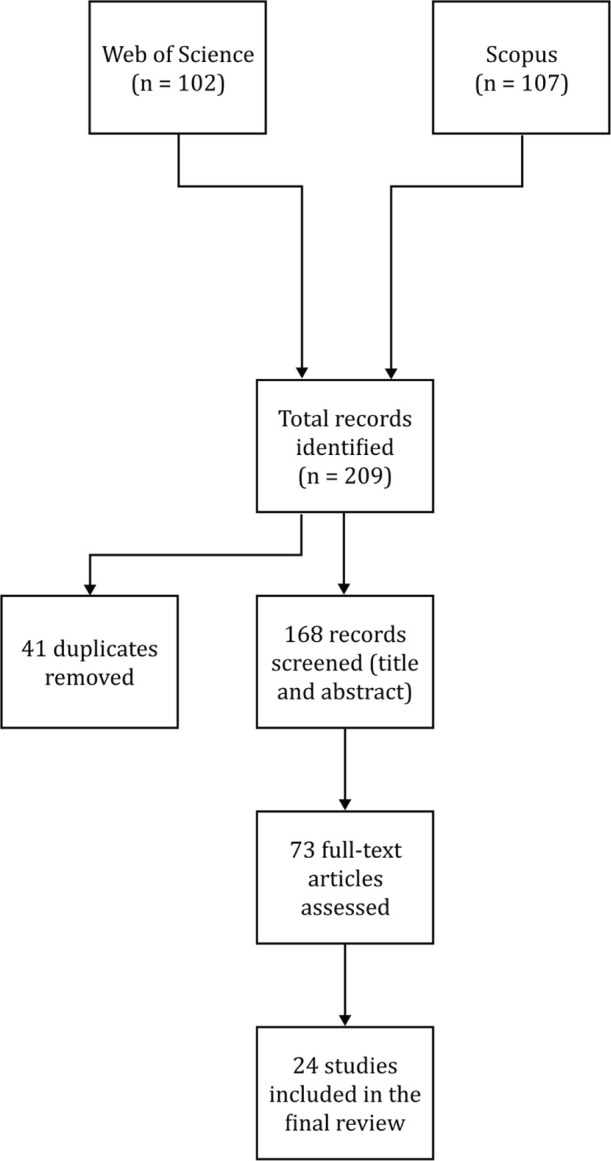
Phases underlying the process of selecting the articles included in the review.

The initial search yielded 209 articles. After the removal of duplicate records, the remaining studies were screened by title and abstract, followed by a full-text review based on a predefined set of eligibility criteria. A total of 95 articles were excluded during the abstract screening phase for not addressing the topic of psychosocial risks at work. An additional 49 articles were excluded during the full-text review because they did not provide a definition of the term. Ultimately, 24 studies met the inclusion criteria and were selected for the final review. The selection process is illustrated in the flowchart presented in Figure 1.

Studies are highly diverse in terms of their objectives (Table 1). They include research assessing psychosocial risks across various populations and professional settings, such as healthcare workers, manufacturing and industrial environments, and academic institutions. Additionally, the review includes articles addressing legislation, standardization, risk assessment, and related interventions as well as studies exploring theoretical and methodological developments on the topic.

**Table 1:** Characteristics of studies included in the review

Authorship	Country	Scope of the journal	Objectives of the study
Abdin et al.^28^	Singapore	Multidisciplinary	Develop and validate the iWorkHealth instrument
Andrade & Falcão^24^	Brazil	Education and sociology	Analyze working conditions, teachers’ perceptions, and psychosocial dimensions; assess psychosocial risk in municipal public school teachers (grades 1–5).
Baç & Ekmekçi^25^	Türkiye	Multidisciplinary	Conduct a psychosocial risk assessment among metal plant maintenance workers using COPSOQ II; compare results with Fine Kinney and AHP methods.
Celestino^22^	Brazil	Nursing	Analyze psychosocial risks in the work of family health nurses and strategies for risk mitigation.
Chirico et al.^32^	Italy, Finland, Netherlands	Public, Occupational, and Environmental Health	Identify countries requiring mandatory employer assessments of psychosocial risks and workplace violence; compare legislative approaches.
Coutinho et al.^27^	Portugal	Public, Occupational, and Environmental Health	Analyze key factors contributing to high exposure to psychosocial risks among hospital workers in Portugal.
De Sio et al.^41^	Italy	Medicine	Analyze the effect of job insecurity (temporary contracts) on the perception of psychosocial risks and vulnerability to work-related stress, with attention to gender differences.
Giménez-Espert et al.^33^	Spain	Public, Occupational, and Environmental Health	The purpose of the study is to examine the perceptions of nurses concerning Covid-19 with respect to such aspects as measures, resources, and impacts on their daily work. The psychosocial risks experienced by these professionals and the relations between these risks and the perception of Covid will also be explored.
Jain et al.^36^	United Kingdom, Ireland, Italy	Public Health	Explore key policies, standards, and frameworks shaping occupational health systems in 12 industrialized countries by using academic and grey literature.
Jespersen & Hasle^30^	Denmark	Engineering	Develop an audit methodology tailored to capturing the specific features of psychosocial risk management.
Lara Satán et al.^26^	Ecuador	Sociology	Demonstrate the need for psychosocial risk management among public transport operators; examine the relationship between psychosocial risks, anxiety, accident rates, and sociodemographics.
Luceño-Moreno et al.^39^	Spain	Clinical Psychology	Validate a model of occupational well-being based on the assessment of perceived psychosocial risk factors.
Luna-Chávez et al.^37^	Mexico	Psychology	Assess workers’ perceptions in a manufacturing industry regarding psychosocial factors related to psychological exhaustion; propose control measures.
Medeiros et al.^23^	Brazil	Nursing	Identify work organization characteristics in the Materials and Sterilization Center and analyze psychosocial risks among nursing staff.
Murillo et al.^31^	Colombia	Occupational Therapy	Identify and propose intervention strategies for occupational psychosocial factors based on occupational therapy.
Navarro et al.^29^	Colombia, Portugal	Computer Science	Propose an intelligent algorithm to improve psychosocial risk projection for use in health promotion and prevention programs.
Nuruzzakiyah et al.^40^	Malaysia	Public Health	Identify psychosocial risks faced by industrial workers.
Ortiz et al.^44^	Colombia	Psychology	Identify occupational psychosocial risk factors among university faculty in Bogotá.
Potter et al.^8^	United Kingdom	Public, Occupational, and Environmental Health	Assess the feasibility, from stakeholders’ perspectives, of advancing policy and practice to improve regulation and mental health in the Australian workforce.
Potter et al.^9^	Australia, United Kingdom	Engineering	Provide a comprehensive resource identifying and assessing policy instruments related to occupational health and safety in Australia, focusing on psychosocial risk management.
Rodrigues et al.^11^	Brazil	Psychology	Analyze the concepts of risk factors and psychosocial risks at work from two prominent theoretical frameworks: the Demand-Control Model and the Psychodynamics of Work.
Seijas-Solano^38^	Venezuela	Public Health	Assess psychosocial risks, work stress, and burnout syndrome among staff at a Venezuelan university for Bioanalysis.
Weissbrodt et al.^35^	Switzerland	Engineering	Evaluate the outcomes of interventions implemented by labor inspectors.
Weissbrodt & Giauque^34^	Switzerland	Engineering	Conduct a systematic review of publications addressing the inclusion of psychosocial risks and the evaluation of the psychosocial work environment in the strategies and practices of labor inspections. The study aims to develop a consistent and transferable evidence framework — using a realist synthesis — to assess the effectiveness of labor inspector visits in preventing psychosocial risks in the workplace.

AHP = analytic hierarchy process; COPSOQ II = Copenhagen Psychosocial Questionnaire.

The analysis of the results revealed a considerable diversity of approaches and objectives in the study of psychosocial risks at work, with notable emphasis on both geographical breadth and interdisciplinary perspectives. The reviewed studies were conducted in various countries — including Brazil, Colombia, Türkiye, Switzerland, the United Kingdom, Italy, and Singapore — reflecting different socio-cultural and occupational contexts. This geographical variety contributes to a broader and more global understanding of the topic; however, it also presents challenges for researchers, particularly in terms of comparing findings and accounting for local cultural specificities.

In addition, the studies were published in journals from a range of academic fields, including Psychology, Public Health, Engineering, and Sociology. This underscores the interdisciplinary nature of psychosocial risk research. Interdisciplinarity allows the phenomenon to be explored from multiple theoretical and practical perspectives, enriching the analysis. At the same time, it demands additional effort to integrate and synthesize results derived from studies grounded in different paradigms and methodologies.

The studies also varied significantly in their focus and target populations. Many examined psychosocial risks within specific professional groups, such as health professionals,^22^,^23^ teachers,^24^ factory workers,^25^ and public transport operators.^26^ Others took a broader approach by assessing working conditions across various sectors and identifying determinants of exposure to psychosocial risk factors, as in the study by Coutinho et al.^27^ in Portugal.

Furthermore, several of the studies reviewed highlight the early stages of conceptualizing and validating new instruments and methodologies for psychosocial risk assessment and prevention. For example, Abdin et al.^28^ developed the iWorkHealth tool, while Navarro et al.^29^ introduced an intelligent algorithm for predicting psychosocial risks. These efforts exemplify the move toward practical, evidence-based tools that can support effective assessment and intervention strategies. Also noteworthy are the studies by Jespersen & Hasle^30^ and Murillo et al.,^31^ which focus specifically on practical interventions and risk mitigation strategies related to psychosocial risks.

However, several limitations are evident across the literature. A key issue is the lack of standardization in the conceptualization of “psychosocial risks.” Many studies do not provide an explicit definition, which compromises the comparability of results and hinders integration across studies. While a targeted focus on specific populations and contexts can be a strength, it may also limit the generalizability of findings — particularly in studies with narrowly defined scopes, such as those focused on national contexts or specific sectors. Finally, the methodological diversity, although valuable, requires careful interpretation. The wide range of instruments and approaches used may have significantly influenced the conclusions drawn in each study.

### ANALYSIS CATEGORIES

Based on the definitions identified in the reviewed studies, seven conceptual categories were established according to the theoretical authors cited in each definition. The results of this categorization are detailed in Table 2. A recurring theme across the definitions is the significant influence of organizational and contextual factors on psychosocial risks. The diversity of perspectives reflects how the concept has evolved and been shaped by different disciplinary frameworks and practical contexts.

**Table 2:** Categories of analysis of the definitions of the concept of psychosocial risks

Authorship of the definition	Authors and year of the articles analyzed	Summary of results
Cox & Griffiths^18^	Potter et al.,^8^,^9^ Coutinho et al.,^27^ Jespersen & Hasle,^30^ Chirico et al.,^32^ Giménez- Espert et al.,^33^ Weissbrodt & Giauque,^34^ Weissbrodt et al.^35^	Psychosocial risks are defined as specific aspects of work design, organization, and management — as well as their social and environmental context — that are likely to cause psychological, social, or physical harm.
International Labour Organization (1984)	Medeiros et al.,^23^ Baç & Ekmekçi,^25^ Abdin et al.,^28^ Jain et al.,^36^ Luna-Chávez et al.,^37^ Seijas-Solano^38^	Psychosocial risks are seen as the interaction between individual and work-related environmental factors. These include elements such as work design, management practices, and the organizational climate, which may negatively impact on workers’ health.
Moreno-Jimenez^19^	Rodrigues et al.^11^ (p. 2), Luceño-Moreno et al.^39^ (p. 67), Murillo et al.^31^ (p. 438)	Psychosocial risk factors are defined as conditions present in a professional context that can negatively impact both the well-being and health of workers, primarily through the generation of stress. These effects may arise from acute or chronic exposure and are associated with harm to physical and mental health.
Leka & Jain^20^	Nuruzzakiyah et al.,^40^ De Sio et al.^41^	Psychosocial risks are described as organizational characteristics and social aspects of the workplace that may harm workers’ health and well-being.^19^
European Union (2010/2011)	Celestino^22^; Lara Satán et al.^26^ (p. 356)	Psychosocial risks are associated with the design, organization, and management of work. They stem from institutional work organization and the interaction with its content. Exposure to these risks can result in physical, mental, or social harm and represents a significant challenge for occupational health and safety. According to EU-OSHA (2010), these risks “arise from deficiencies in the design, organisation and management of work, as well as from a problematic social context of work, which can have negative psychological, physical and social effects such as work-related stress, exhaustion or depression.”
Sauter & Murphy,^42^ Sauter et al.^43^	Navarro et al.^29^ (p. 2), Ortiz et al.^44^ (p. 3)	Psychosocial risks are viewed as stressors capable of altering and destabilizing an individual’s capacity to manage work demands, thereby negatively affecting psychological health.^42^ Prolonged exposure to certain work conditions may lead to persistent psychobiological activation and associated health consequences.^43^
Karasek^45^	Andrade & Falcão^24^ (p. 706)	Based on the JCQ, proposed by Karasek,^45^ psychosocial risk is understood as a situation involving high psychological demands (e.g., workload, pace, complexity) combined with low decision latitude (i.e., limited control over one’s tasks and limited opportunity for skill use and development).

JCQ = Job Content Questionnaire.

Cox & Griffiths^18^ were the most frequently cited authors, referenced in eight studies, including those by Chirico et al.,^32^ Coutinho et al.,^27^ Giménez-Espert et al.,^33^ Jespersen & Hasle,^30^ Potter et al.,^8^,^9^ Weissbrodt & Giauque,^34^ and Weissbrodt et al.^35^ They define psychosocial risks as specific aspects of work design, organization, and management — alongside the social and environmental context of work — that have the potential to cause psychological, social, or physical harm. Their approach highlights the various ways in which organizational and social conditions may affect workers’ health and well-being.

The definition proposed by the ILO^10^ was cited in six studies, including those by Abdin et al.,^28^ Baç & Ekmekçi,^25^ Jain et al.,^36^ Luna-Chávez et al.,^37^ Medeiros et al.,^23^ and Seijas-Solano.^38^ This perspective emphasizes the interaction between individual and organizational factors within the work environment — such as work design, management practices, and organizational climate — as key elements in the development of risks to workers’ health.

Moreno-Jiménez^19^ is cited in three studies: Rodrigues et al.,^11^ Luceño-Moreno et al.,^39^ and Murillo et al.^31^ His definition frames psychosocial risks as working conditions that may negatively affect workers’ health and well-being, particularly through stress. This perspective emphasizes the cumulative effects of both acute and chronic exposure to adverse workplace factors.

Leka & Jain^20^ are referenced in two studies: Nuruzzakiyah et al.^40^ and De Sio et al.^41^ Their definition focuses on the organizational and social aspects of the workplace, asserting that psychosocial risks originate from these structural elements and can pose significant hazards to workers. This approach adopts a more targeted view of how organizational structures directly influence worker health.

Celestino et al.^22^ and Lara Satán et al.^26^ adopt the definition provided by the European Union, which links psychosocial risks to deficiencies in the design, organization, and management of work. This definition highlights the potential for physical, mental, and social harm, framing these risks as central challenges to occupational health.

Two studies — Navarro et al.^29^ and Ortiz et al.^44^ — reference the work of Sauter & Murphy^42^ and Sauter et al.,^43^ defining psychosocial risks as stressors that impair workers’ capacity to manage work demands. This condition may lead to sustained psychobiological activation, with harmful consequences for health. This perspective contributes an important psychobiological dimension to the broader understanding of psychosocial risks.

Finally, Karasek’s^45^ demand-control model is referenced by Andrade & Falcão,^24^ linking psychosocial risks to work situations characterized by high psychological demands combined with low decision latitude. This theoretical framework is widely recognized for identifying specific organizational conditions that intensify psychosocial risk exposure.

Collectively, these varied definitions illustrate how the concept of psychosocial risks has evolved and expanded over time, incorporating contributions from organizational analysis, individual factors, and psychobiological perspectives. However, this conceptual diversity also underscores a key challenge: the need for a more standardized and operational definition that can be consistently applied in both research and practical interventions. Achieving greater definitional clarity is especially important for enabling international comparisons and supporting the effective development of occupational health policies.

## DISCUSSION

The main objective of this study was to investigate how psychosocial risks at work are defined in scientific publications, based on the analysis of 24 articles published between 2017 and 2021. The findings reveal a notable conceptual variety, which reflects both theoretical progress and the ongoing methodological and practical challenges within the field. This plurality highlights the absence of a definitive consensus regarding the concept of psychosocial risks — an issue of particular importance for both academic research and occupational health practice.

Definitions of psychosocial risks have evolved over several decades, although some foundational perspectives were established early on — particularly by the ILO,^10^ which emphasized the interaction between individual and organizational factors within the work environment. Pioneering models by Karasek^45^ and Cox & Griffiths^18^ also focused on organizational dimensions, highlighting objective aspects of working conditions such as job demands, control, and the design and management of work. As the field has progressed, definitions have become increasingly complex, incorporating subjective and contextual elements. More recent approaches include workers’ perceptions, levels of social support, and broader socio-political influences. This evolution reflects the growing recognition of the multifaceted nature of psychosocial risks and occupational stress.^19^,^20^

Another key point identified in this review is the often-implicit distinction between psychosocial risks and psychosocial risk factors. While the former refers to the likelihood that certain working conditions may negatively impact workers’ health, the latter corresponds to the specific elements or characteristics of the work environment that give rise to those conditions.^46^ Although this distinction is conceptually essential, it is frequently overlooked in literature. Recent standards, such as ISO 45003,^21^ seek to address this gap by providing a clear framework for defining and managing psychosocial risks. Nonetheless, the absence of a widely accepted definition continues to hinder result comparability and limits organizations’ ability to implement effective interventions. This review therefore underscores the urgent need for a clear, integrated, and operational definition of psychosocial risks. In response, we propose a refined integrative definition informed by both classic and contemporary theoretical references — including Cox & Griffiths,^18^ Karasek,^45^ Moreno-Jiménez,^19^ and Leka & Jain,^20^ — as well as by recent normative guidelines, particularly ISO 45003.^21^

Psychosocial risks at work refer to situations that arise from the way a job or work process is designed, managed, or socially experienced. These risks are closely linked to individual perceptions and workplace interactions and can lead to measurable negative outcomes such as stress, burnout, anxiety, depression, or interpersonal conflict. In contrast, psychosocial risk factors are specific conditions within the work environment — such as excessive workload, time pressure, poorly defined roles, job insecurity, or lack of support — that increase the likelihood of psychosocial risks manifesting. Conversely, protective factors — such as organizational support, autonomy, and role clarity — can mitigate workers’ vulnerability to these risks. The absence of such protective factors can, in turn, heighten the probability of adverse psychosocial outcomes.

This definition addresses previous limitations by simultaneously emphasizing both objective and subjective aspects of psychosocial risks, while explicitly recognizing the importance of workers’ perceptions. To translate this definition into effective practice, it is essential to follow the key phases of a structured process for assessing and improving health and safety conditions at work — such as those outlined in ISO 45001. Specifically, we recommend the following:

1. Conducting a multidimensional assessment: Organizations should employ validated quantitative tools such as the Copenhagen Psychosocial Questionnaire (COPSOQ III), Job Content Questionnaire (JCQ), and iWorkHealth, while also incorporating qualitative methods like semi-structured interviews and focus groups. In addition, data not captured by these instruments — such as absenteeism and turnover rates — should be collected. This step provides comprehensive insight into both the objective and subjective dimensions of psychosocial risk exposure.
2. Classifying and prioritizing the risks: Identified risks should be classified according to their frequency, severity, and likelihood of causing harm. Tools such as risk matrices and multicriteria analysis can support a systematic and transparent prioritization process, thereby informing decision-making in psychosocial risk management.
3. Developing and implementing tailored interventions: Interventions should be strategically designed in response to the specific risks identified. These may include managerial training in social support practices, adjustments in work design to better align with individual needs, initiatives to promote work-life balance, and solutions for emerging challenges related to digital innovation and the evolution of remote work arrangements.
4. Longitudinal evaluation and ongoing review: Finally, organizations should engage in ongoing evaluation of intervention effectiveness through periodic assessments using both quantitative and qualitative methods. Regular monitoring and iterative adjustments ensure that actions remain sustainable and aligned with evolving organizational and contextual dynamics.

Despite the contributions presented, several limitations must be acknowledged. One limitation concerns the use of specific keywords, which may have constrained the literature search and excluded studies that approach the topic from different conceptual or linguistic angles. Additionally, the selection of the 2017–2021 timeframe aimed to capture recent contributions aligned with current transformations in the world of work. While this may have excluded relevant studies published before or after this period, its impact is mitigated by the strong alignment of the selected articles with ongoing debates and pressing issues in the field. A more significant limitation is the lack of an in-depth analysis of practical interventions associated with the definitions reviewed. Such an analysis would have been valuable to better connect theoretical developments with applied strategies in organizational settings.

Future research should address these limitations by adopting more inclusive and methodologically robust approaches. Broader search strategies — using a wider variety of terms and multiple databases — can help ensure a more representative sample of publications. In addition, extending the review period may capture historical shifts and long-term trends in definitions and conceptual approaches. Promising avenues for future inquiry include examining how psychosocial risks are perceived and managed across different cultural and economic contexts. Special attention should also be given to emerging risks related to digitalization, technological change, and evolving models of work. Furthermore, longitudinal and comparative studies are needed to explore the long-term effects of psychosocial risks, as well as the contextual factors that mediate their impact on health and productivity.

Moreover, future research should prioritize the testing and adaptation of standardized assessment instruments — such as those outlined in ISO 45003 — to ensure their applicability across diverse cultural settings and organizational contexts. The integration of complementary qualitative and quantitative methods is essential to deepen understanding of the independent and dependent variables that influence psychosocial risks. Finally, the development of practical guidelines is necessary to translate conceptual definitions into actionable strategies. Such guidelines will support the implementation of targeted organizational interventions, helping to ensure that theoretical advancements are aligned with the practical demands and realities of contemporary occupational health.

## CONCLUSIONS

This study conducted an integrative review of current definitions of psychosocial risks at work, revealing significant conceptual diversity and underscoring the need for a clearer and more operational understanding of the concept. The lack of consensus not only limits the comparability of research findings but also hinders the development of effective organizational policies and interventions. The integrative definition proposed in this article offers a practical and comprehensive conceptual framework by clearly distinguishing between psychosocial risks, risk factors, and protective factors. This clarity provides a solid foundation for both assessment and intervention in workplace settings. We encourage future research to further explore emerging psychosocial risks, particularly in the context of digital transformation and evolving work models. Aligning these efforts with international standards (e.g., ISO 45003) will support theoretical and practical advancements, contributing to healthier, more sustainable work environments and ultimately enhancing workers’ occupational health and well-being.
